# Endoscopic resection of giant lipoma mimicking colonic neoplasm initially presenting with massive haemorrhage: a case report

**DOI:** 10.1186/1757-1626-2-6462

**Published:** 2009-03-10

**Authors:** Georgia Lazaraki, Dimitrios Tragiannidis, Persefoni Xirou, Andreas Nakos, Ioannis Pilpilidis, Ioannis Katsos

**Affiliations:** 1 Department of Gastrointestinal Oncology, Theagenion Cancer Hospital, Al. Simeonidi 2 str, 54007, Thessaloniki, Greece; 2 Department of Pathology, Theagenion Cancer Hospital, Al. Simeonidi 2 str, 54007, Thessaloniki, Greece

## Abstract

Lipomas of the colon are benign tumors that rarely occur. Their size ranges from 2 mm to several cm. They are usually asymptomatic but occasionally they present with clinical manifestations depending on tumor size, localization and complications, which often lead to diagnostic difficulty. A 40-year-old man presented with massive rectal haemorrhage. During colonoscopy a giant polyp of over 50 mm in its bigger diameter, with a thick stalk of 2 cm, located in the transverse colon, was revealed. Endoscopic resection was performed with success. Histologic examination demonstrated a giant lipoma. In this report discussion over endoscopic resection of colonic lipomas mimicking neoplasms is also performed.

## Introduction

Lipomas of the colon are benign tumors with extremely low malignant potential that occur rarely. They are the second most common benign colonic tumor after adenomatous polyps [1]. Although the majority remain asymptomatic, colonic lipomas may present with symptoms such as pain, diarrhoea, obstruction, and bleeding. Colonic lipomas size ranges from 2 mm to 30 cm and may occasionally mimic colonic malignancies. Size (>2 cm) appears to correlate with symptoms and 75% of patients with a lesion larger than 4 cm have symptoms [2] and in this case they should be resected either endoscopically or surgically [1,3].

We report here a case of a giant colonic lipoma with the endoscopic appearance of neoplasmatic polyp, successfully removed in a single piece.

## Case presentation

**Figure 1:**
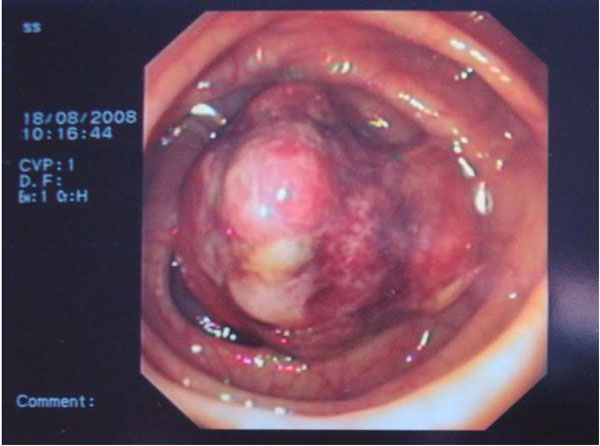
**The polyp of the transverse colon responsible for the massive hemorrhage**.

A 40-year-old Caucasian man from Greece presented to the emergency room of a hospital with massive rectal bleeding and consequent anemia. *The patient reported more than 15 loose bowel movements mixed with blood during last 24 hours.* Physical examination was unremarkable. The patient denied any changes in bowel habits frequency with an average of 1-2 bowel movements per day. He reported no previous medical history and took no medications. Results of laboratory investigation including carcinoembryonic antigen (CEA) were within normal limits. Colonoscopy revealed a large polyp (>5 cm diameter) in the transverse colon (Figure 1). The lesion appeared to be pedunculated with a thick stalk of approximately 2 cm length and ~1.5 cm diameter. The overlying mucosa was ulcerated and the lesion was soft and compressible. Suspicion of a large adenomatous polyp with malignant potential was raised. Biopsies from the lesion suggested necrotic tissue without malignant tissue and the patient was referred to our department for polypectomy.

**Figure 2:**
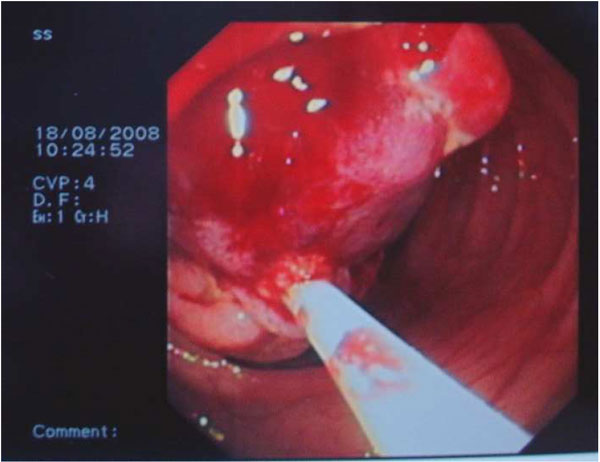
**The polyp was ensnared in the basis of the stalk with a large exagonal snare of 60 mm loop**.

**Figure 3:**
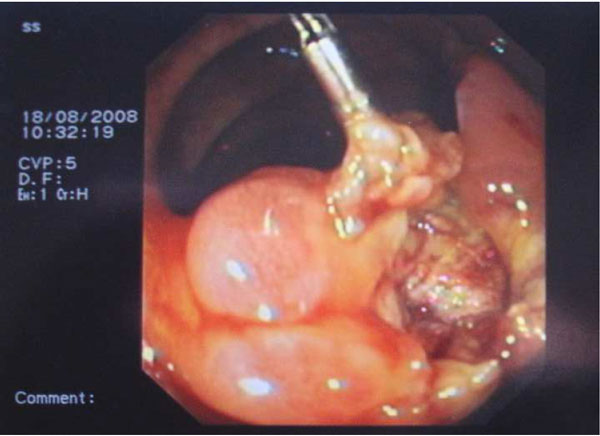
**The ulcer crater left in the basis of the stalk after the polyp excision**.

**Figure 4:**
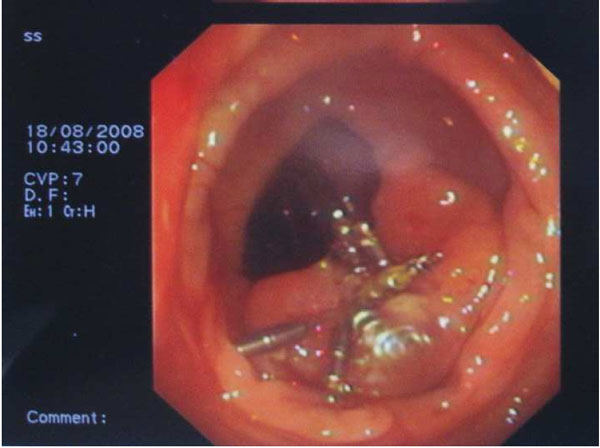
**Endoclips were placed to approach the ulcer margins**.

The patient was informed for increased risk of perforation and bleeding during endoscopic removal and an informed consent was obtained. A large hexagonal electrosurgical snare of 60 mm loop (Olympus, Tokyo, Japan) was needed to ensnare the lesion near its base (Figure 2). Subsequent excision of the lesion in a single piece was able to be performed with the use of electrosurgical monopolar current (Figure 3). Finally, endoclips were placed to approach the ulcer margins (Figure 4). No procedure-related complications occurred and the patient was released 24 hours later. Histological examination revealed characteristic lipoma of colon.

## Discussion

This was the seventh case of giant colonic lipoma in our department and the second in this series initially presenting with massive hemorrhage. In the Mayo Clinic series, 46% of large-bowel lipomas were discovered incidentally in specimens removed for other diseases, 11% were resected because of a neoplasm suspected of being a carcinoma, and 6% were symptomatic [3]. The lesion did not have the "classic" endoscopic features of colonic lipomas ("tenting sign"- grasping the overlying mucosa, "cushion sign"-flattening and restoration of the shape of the lipoma, "naked fat sign"-extrusion of fat after biopsy of the colonic mucosa) rather mimicking endoscopically an adenomatous polyp. Colonoscopy is reliable for the diagnosis of a typical lipoma, but it may prove to be of no help when the lesion is atypical [3]-[5].

Pathologically, lipomas are well-differentiated tumors arising from deposits of adipose connective tissue in the bowel. Sarcomatous changes, in colonic lipomas have not been reported, but intermittent torsion and relative ischemia can give rise to a pseudomalignant appearance. The greatest clinical significance of lipomas lies in their potential to be confused with adenomatous polyps or other aggressive pathology [6]. Despite recent diagnostic innovations it has been reported that the preoperative diagnostic accuracy is only about 62%. Therefore, in most cases, histological diagnosis is arrived only after excision of the tumor. This case demonstrates how, on rare occasions, large colonic lipomas and malignancies can be difficult to differentiate prior to resection. With regards to symptoms and endoscopic appearance, the two can be indistinguishable. Even with abdominal imaging and direct colonoscopic visualization, lipomas can imitate neoplasms [7].

The decision whether to remove lipomas and which method is the best option, either endoscopically or surgically remains controversial. Because the majority of lipomas are submucosal, endoscopic removal entails high risk of morbidity due to perforation compared with adenomatous polyps, since its high water content requires a tremendous amount of heat to cut through the lipoma. Endoscopic polypectomy of large lipomas is difficult, although possible in selected cases (especially if pedunculated) as it both is reported in the literature [2,3,6,8]-[12] and observed in case-series of our centre [13]. In the series by Pfell et al [6], 3 of 7 patients had a subsequent perforation after endoscopic removal of colonic lipomas. Surgery remains an option, especially for large colonic lipomas. However, endoscopic removal would be preferable over surgical excision if it can be done safely. In more recent endoscopic series, various techniques such as saline injection assisted polypectomy and the use of endoscopic ultrasound had been advocated to reduce the risk of perforation [8,10]. On the other hand, conventional open colonic resection is considered a major undertaking with considerable morbidity, especially in elderly patients with coexisting medical conditions. Recently, laparoscopic colonic resection has been shown to be associated with less postoperative pain, shorter duration of ileus, and quicker recovery; therefore, it is recommended for the treatment of benign colorectal conditions such as large polyps [14,15]. In our case, a detailed endoscopic examination of the base of the lesion was performed as suggested [8] and a stalk of 2 cm was demonstrated. As noted by Christie et al [9] and Stone et al [16], removal of truly pedunculated lipomas does not provide any increased risk as compared with the removal of any pedunculated adenomatous type polyp. However, it has been reported, that in some cases, what it is considered to be a stalk is rather a pseudopedicle caused by serosal invagination, which might include the muscularis propia and the serosal layers, and in this case cutting could be disastrous [17].

In conclusion, it should be noted that colon lipomas, although rare, should be considered in the differential diagnosis of large bowel tumors. Endoscopic approach remains a safe and effective option for giant lipoma resection, provided each case is selected carefully and procedures are performed by skilled endoscopists in centres with experience.

## Consent

Written informed consent was obtained from the patient for publication of this case report and accompanying images. A copy of the written consent is available for review by the Editor-in-Chief of this journal.

## Competing interests

The author(s) declare that they have no competing interests.

## Authors' contributions

GL is a senior gastroenterologist in our department, performed the polypectomy, and was the major contributor in writing the manuscript. DT collected the patient's data and assisted in the procedure. PX performed the histological examination of the polyp, and was a major contributor in writing the manuscript. AN was responsible for all the artwork seen in the manuscript. IP contributed in writing the manuscript. IK is the head of the department and revised the manuscript.
